# Cholinesterase and Inflammation: Exploring Its Role and Associations with Inflammatory Markers in Patients with Lower Extremity Artery Disease

**DOI:** 10.3390/biomedicines13040823

**Published:** 2025-03-30

**Authors:** Maximilian Mitteregger, Sabine Steiner, Andrea Willfort-Ehringer, Thomas Gremmel, Renate Koppensteiner, Michael Gschwandtner, Eva-Luise Ritter-Hobl, Christoph W. Kopp, Patricia P. Wadowski

**Affiliations:** 1 Department of Internal Medicine II, Division of Angiology, Medical University of Vienna, 1090 Vienna, Austria; maximilian.mitteregger@oegk.at (M.M.); sabine.m.steiner@meduniwien.ac.at (S.S.); andrea.willfort-ehringer@meduniwien.ac.at (A.W.-E.); renate.koppensteiner@meduniwien.ac.at (R.K.); michael.gschwandtner@meduniwien.ac.at (M.G.); christoph.kopp@meduniwien.ac.at (C.W.K.); 2 Cardiology, Coronary Care Unit and Angiology, Department of Internal Medicine II, Hanusch Krankenhaus, 1140 Vienna, Austria; 3 Department of Internal Medicine I, Cardiology and Intensive Care Medicine, Landesklinikum Mistelbach-Gänserndorf, 2130 Mistelbach, Austria; thomas.gremmel@mistelbach.lknoe.at; 4 Institute of Cardiovascular Pharmacotherapy and Interventional Cardiology, Karl Landsteiner Society, 3100 St. Pölten, Austria; 5 Karl Landsteiner University of Health Sciences, 3500 Krems an der Donau, Austria; 6 Department of Clinical Pharmacology, Medical University of Vienna, 1090 Vienna, Austria; eva-luise.ritter-hobl@meduniwien.ac.at

**Keywords:** inflammation, cholinesterase, lower extremity artery disease, peripheral artery disease, neutrophil-to-lymphocyte ratio, platelet-to-lymphocyte ratio, critical ischemia, obesity

## Abstract

**Background:** Inflammation is a major driver of atherosclerotic diseases including lower extremity artery disease (LEAD). Serum cholinesterase (ChE) has been shown to impact cardiovascular health and regulate inflammatory processes. **Objectives:** The aim of this study was to investigate the relationship between serum ChE levels and inflammatory markers in patients with hemodynamically relevant iliac artery stenosis, assessing its potential role in the inflammatory processes of lower extremity artery disease (LEAD). **Methods:** In the following retrospective data analysis, we investigated 150 patients with hemodynamically relevant iliac artery stenosis as documented by a delta peak systolic velocity (δPSV) ≥ 1.4 m/s and investigated the possible influence of ChE on established inflammatory markers, such as neutrophil-to-lymphocyte ratio (NLR), platelet-to-lymphocyte ratio (PLR) and hemoglobin-to-platelet ratio (HPR), along with other routine laboratory or vascular parameters. **Results:** ChE levels differed significantly between patients with stable claudication (Fontaine stage II) and critical ischemia (Fontaine stages III and IV): 7.76 mg/dL (6.55–8.7 mg/dL) vs. 6.77 mg/dL (5.85–7.48 mg/dL), *p* = 0.004. Using the spearman correlation coefficient, testing of NLR and ChE revealed a highly significant inverse correlation, with a coefficient of −0.303 (*p* < 0.001). Additionally, a weak inverse correlation was observed between PLR and ChE, with a coefficient of −0.162 (*p* = 0.049). Patients with an elevated body mass index (BMI) showed increased levels of serum ChE, with a spearman correlation coefficient of 0.298 (*p* < 0.001). **Conclusions:** The observed correlations in this study depict active inflammation in LEAD with an emphasis on patients with critical ischemia. Serum ChE could serve as a potential biomarker for inflammation in patients with LEAD, particularly in distinguishing between stable claudication and critical ischemia. Future research is needed to explore the role of ChE as a complementary biomarker, offering insights into the cholinergic regulation of inflammation in LEAD.

## 1. Introduction

### 1.1. PAD

Peripheral arterial disease (PAD) is characterized by stenosis or occlusion of a target vessel, due to the mechanisms of atherosclerosis, thrombotic or embolic events [1]. It is a highly prevalent disease with a noticeable age dependency, with prevalence increasing to 15–20% from the age of 70 [2]. Furthermore, the presence of lower extremity arterial disease (LEAD) has been shown to put patients at a significant risk of developing other cardiovascular diseases such as coronary artery disease (CAD), myocardial infarction (MI) and cerebrovascular disease (CVD) [3,4]. Yet, patients with LEAD are vastly underdiagnosed and undertreated in comparison to patients with CAD [5,6,7]. Established risk factors for patients with LEAD include diabetes mellitus [odds ratio (OR) 1.5], chronic kidney disease (CKD) (OR 2.0), hypertension (OR 1.5), hypercholesterolemia (OR 1.3), obesity (OR 1.0) and smoking (OR 4.1) [8]. LEAD is an inflammatory disease and the primary factor contributing to its pathology is atherosclerosis, which is characterized by an endothelial dysfunction and the process of plaque formation and destabilization [9,10,11,12,13]. It has been demonstrated that patients affected by limb ischemia show high levels of inflammation and have an increased mortality rate [14]. The wide range of treatment options for patients with LEAD range from optimizing cardiovascular risk factors, including lifestyle intervention, antithrombotic therapy and invasive strategies like endovascular or surgical revascularization [15]. Nevertheless, the main challenge associated with endovascular revascularization remains the occurrence of restenosis [16]. Patients with LEAD are at increased risk of critical limb ischemia (CLI) and major adverse limb events (MALE), like amputation further decreasing the quality of life [17].

### 1.2. Cholinesterase

Parasympathetic dysfunction has been linked to poor cardiovascular outcomes. In support of this, indicators of cardiovascular parasympathetic dysfunction, in the means of elevated resting heart rate, reduced heart rate response during exercise and delayed heart rate recovery after exercise, have all been demonstrated to independently predict adverse cardiovascular outcomes [18,19,20,21]. In patients undergoing coronary angiography a low serum level of cholinesterase (ChE) was linked to the development of major adverse cardiac events (MACE) [22]. In another study, low ChE was a significant predictor of mortality in patients with stable CAD [23]. Both increased and decreased serum ChE levels appear to influence cardiovascular health. A study reported that ChE levels were significantly elevated in patients with severe myocardial ischemia with three-vessel CAD [24]. The same elevation in serum ChE, as a recent study figured out, has been found in patients with stable CAD, where the number of diseased vessels increased significantly with serum ChE levels [25]. Gremmel et al. reported the association of low ChE and the increased risk of long-term adverse ischemic events in patients undergoing primary unilateral angioplasty with self-expanding bare metal stent implantation for superficial femoral artery (SFA) stenosis [26]. The above-mentioned findings support the image of a U-shaped curve when interpreting the influence of ChE and cardiovascular risk.

**Figure 1:**
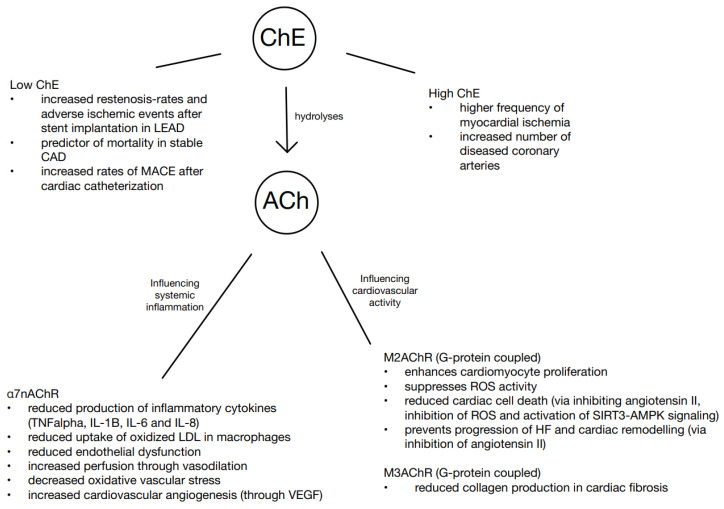
ChE influencing inflammation and cardiovascular diseases. α7nAChR, alpha 7 nicotinic acetylcholine receptor subunit; Ach, acetylcholine; CAD, coronary artery disease; ChE, cholinesterase; HF, heart failure; IL-1B, interleukin 1B; IL-6, interleukin-6; IL, interleukin-8; LDL, low-density lipoprotein; LEAD, lower extremity artery disease; MACE, major adverse cardiac events; M2AChR, type 2 muscarinic acetylcholine receptor; M3AChR, type 3 muscarinic acetylcholine receptor; ROS, reactive oxygen species; SIRT3-AMPK, sirtuin 3/AMP-activated protein kinase; TNFα, tumor necrosis factor alpha; VEGF, vascular endothelial growth factor.

The cholinergic anti-inflammatory pathway (CAP) was first described in 2000. It is a parasympathetic anti-inflammatory signaling cascade that can modulate inflammatory processes [27,28,29]. Furthermore, serum acetylcholine (ACh) reduces the production of inflammatory cytokines (TNF-α, IL-1β, IL-6, and IL-18) through the alpha 7 nicotinic acetylcholine receptor subunit (α7nAChR) hereby preventing systemic inflammatory response syndrome (SIRS) development [27,30,31]. One step in plaque formation that ACh can reduce via signaling is the uptake of low-density lipoprotein (LDL) into macrophages [32]. Another way ACh can influence cardiovascular disease is through a reduction in endothelial dysfunction and the promotion of vasodilation, thereby enhancing tissue perfusion [33]. In addition, oxidative vascular stress is reduced via α7nAChR [34] and coronary perfusion is improved in regard of cardiac angiogenesis through vascular endothelial growth factor (VEGF) [35]. Exercise enhances myocardial type 2 muscarinic acetylcholine receptor (m2AChR) protein expression, which is also necessary in the prevention of myocardial apoptosis [36]. In a study, it was demonstrated that ACh and vagal stimulation can protect the heart to a certain degree from ischemic events, with a reduced production of reactive oxygen species (ROS) [37]. It has been observed that ACh prevents cell apoptosis by inhibiting the effect of angiotensin II and by inhibiting the production of ROS through activation of sirtuin 3/AMP-activated protein kinase (SIRT3-AMPK) signaling [38,39]. In experiments such as vagal nerve stimulation in heart failure, ACh was able to prevent the progression of heart failure and cardiac remodeling via the inhibition of angiotensin II [40,41,42]. When ACh binds onto the type 3 muscarinic acetylcholine receptor (m3AChR), which is being expressed on cardiac fibroblasts, it can influence cardiovascular disease in terms of reducing collagen production in cardiac fibrosis [43] (Figure 1).

Considering that LEAD, especially in comparison to CAD is still widely underdiagnosed and undertreated [44], ChE may function as a surrogate parameter for identifying high-risk patients who would benefit from intensified treatment options [26]. However, the relationship between ChE and inflammatory parameters such as NLR, PLR and HPR in patients with LEAD remains unclear. The present study therefore assessed a possible correlation between these parameters.

### 1.3. PLR, NLR, HPR

In the last years, there has been an increased scientific interest in finding novel inflammatory markers predicting cardiovascular risks. Neutrophil-to-lymphocyte ratio (NLR) and platelet-to-lymphocyte ratio (PLR) serve as biomarkers for inflammation and given the simplicity in their calculation, it could be advantageous to use them more frequently in the assessment of ischemic vascular risk profiles in patients [45,46,47]. It has been observed that there is a correlative relationship among NLR, PLR and the occurrence of CLI in patients with LEAD [48,49]. Lee et al. recently published the predictive value of NLR and PLR regarding target vessel restenosis (TVR) following infrainguinal angioplasty [50]. Moreover, it was found out that elevated NLR and PLR values are associated with an 11 times higher increase in the risk of amputation and a 22 times higher increase in the risk of mortality in patients with acute limb ischemia [51].

Hemoglobin-to-platelet ratio (HPR) and platelet-to-hemoglobin ratio (PHR), the inverse of HPR, are emerging markers with a limited amount of published literature. PHR has recently attracted considerable interest as a parameter with potential prognostic value in cardiovascular disease [52,53,54,55]. In terms of PAD, there has been a study by Ozbeyaz et al., which included patients who underwent peripheral angiography on account of below-knee CLI, who were not found suitable for endovascular and surgical revascularization. They have demonstrated that PHR was a reliable predictor of below-knee amputations [56].

## 2. Materials and Methods

This study was conducted as an exploratory retrospective data analysis at the Department of Internal Medicine II, Division of Angiology, Medical University of Vienna. The Ethics Committee of the Medical University of Vienna approved the study in June 2022 with the number 1442/2022. Part of this study was published as a thesis of Dr. med. univ. Maximilian Mitteregger [57].

We included 150 patients who were admitted to the Department of Internal Medicine II, Division of Angiology, and diagnosed with LEAD and a hemodynamically relevant stenosis [delta peak-systolic-velocity (δPSV) ≥ 1.4 m/s] measured by color-coded duplex ultrasonography of the common or external iliac artery between 1 January 2016 and 31 December 2018. This was an exploratory pilot analysis. Therefore, we did not perform a sample size calculation. The results should be regarded as hypothesis-generating. A possible influence of ChE on established inflammatory markers, such as PLR, NLR and HPR, along with other routine laboratory or vascular parameters was investigated. Due to outliers in the routine laboratory data and skewed distributions, we decided to use only non-parametric tests.

Blood samples were drawn during the first 24 h after admission by aseptic venipuncture and the blood parameters were measured in the central laboratory of the Medical University of Vienna according to standardized protocols. Butyrylcholinesterase was measured by the central laboratory using Roche Cobas 8000 (Roche Diagnostics, Vienna, Austria). Hemoglobin, thrombocytes, neutrophil granulocytes and lymphocytes were measured on the Sysmex XN1000 (Sysmex Austria, Vienna, Austria).

Exclusion criteria were underlying hematological diseases, such as leukemia, von Willebrand disease, polycythemia vera, immune thrombocytopenia, active bleedings and heparin-induced thrombocytopenia (HIT).

Baseline characteristics are presented as median (interquartile range) for continuous variables and as number (%) for nominal variables. Spearman rank correlation was performed to assess correlations of ChE and other routine laboratory or vascular parameters. Two-sided *p*-values < 0.05 were considered the threshold for statistical significance. For data collection, the variables required for the study were collected from the Medical University of Vienna Information Management (AKIM) and analyzed using the IBM SPSS Statistics Version 29 (IBM Corp, Armonk, NY, USA).

## 3. Results

A total of 150 patients were included in the study. Characteristics of the study population depicted for patients with stable claudication and critical ischemia are given in Table 1. Patients with critical ischemia were significantly older than patients with stable claudication (*p* < 0.001). CAD differed significantly between patients with claudication and those with critical ischemia (*p* = 0.005). In addition, patients with critical ischemia were significantly more likely to have chronic kidney disease (*p* = 0.007). No significant difference could be analyzed for diabetes mellitus, smoking, hypertension, obesity and hyperlipidemia. Patients with critical ischemia were significantly more likely to be prescribed lipid-lowering drugs and diuretics.

**Figure 2:**
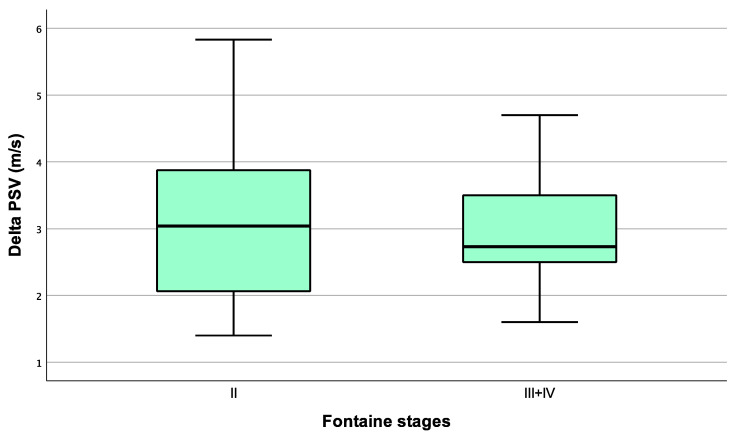
δPSV (difference in peak systolic velocity; m/s) in patients with stable claudication and critical ischemia.

The parameter δPSV is used for the purpose of determining the degree of stenosis. A value of 1.4 or above is considered to indicate a hemodynamically relevant stenosis. The median δPSV in the study population with claudication was 3.04 (2.03–3.9), and in those with critical ischemia it was 2.73 (2.38–3.9), *p* = 0.824 (Figure 2).

The study population included patients with at least one stenosis of the common or external iliac artery. LEAD was categorized in accordance with the Fontaine classification. Subsequently, a more detailed analysis of the frequency distribution in the study population revealed that 111 patients had Fontaine stage IIb. This corresponds to 74% of the patient population. In total, 20 patients suffered from Fontaine stage IV, the most severe form of LEAD, which is associated with necrosis and gangrene. Only two (1.3%) patients in the study population were asymptomatic and, therefore, Fontaine stage I. Fontaine stage IIa was recorded in 12 (8%) patients. According to the patient files, only five (3.3%) patients were categorized as Fontaine stage III.

Cholinesterase levels differed significantly between Fontaine stages II and IV (*p* = 0.001), as well as between patients with stable claudication (Fontaine stages II) and those with critical ischemia (Fontaine stages III and IV); see Table 2. Since there were only two patients with Fontaine stage I, data of those are not depicted.

The levels of PLR, NLR and HPR were 132.1 (98.34–175.06), 2.8 (2.11–4.06) and 0.05 (0.04–0.07), respectively, in the overall study population.

**Figure 3:**
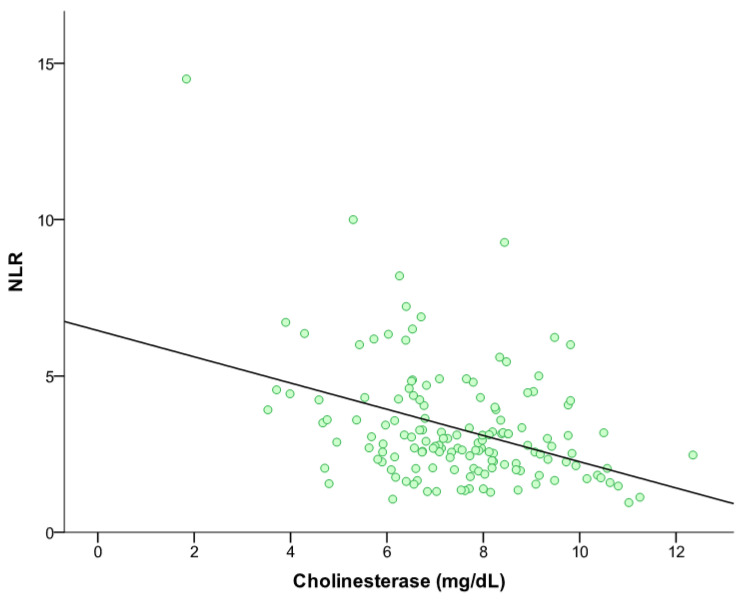
Correlation between NLR and cholinesterase (r= −0.303, *p* < 0.001).

In the next step, the correlations between the inflammatory markers PLR, NLR, HPR and ChE were tested using the spearman correlation coefficient with two-sided significance. A weak inverse correlation was identified between PLR and ChE, with a spearman correlation coefficient of −0.162 and a *p*-value of 0.049. Furthermore, the correlation test of NLR and ChE yielded a highly significant inverse correlation, with a spearman correlation coefficient of −0.303 and a *p*-value of <0.001, Figure 3.

No significant correlation was observed between HPR and the ChE. A correlation coefficient of 0.122 and a *p*-value of 0.140 were measured.

A further objective was to investigate whether a correlation between δPSV and PLR, NLR and HPR could be measured. The spearman correlation coefficient between δPSV and PLR was −0.26, with a *p*-value of 0.750. This indicates that no correlation is present between these two parameters. The correlation test between δPSV and NLR was also negative, with a correlation coefficient of −0.35 and a *p*-value of 0.668. Similarly, the analysis of δPSV and HPR demonstrated no correlation. A correlation coefficient of 0.081 and a *p*-value of 0.325 were calculated for δPSV and HPR; see Table 3.

In addition, it was part of the study to analyze possible correlations between ChE and δPSV, age, BMI, thrombocytes, leucocytes, CRP and fibrinogen. The correlation between ChE and BMI was significant with a spearman correlation coefficient of 0.298 and a *p*-value of <0.001; see Table 4.

In a further step, we investigated possible differences of the main variables and clinical characteristics between men and women; see Table 5.

When analyzing the age difference between men and women, it appeared that women were significantly older than men (*p* ≤ 0.001). In women, there was a significant inverse correlation between PLR and ChE (r = −0.293, *p* = 0.026), which was not present in men (r = −0.087, *p* = 0.414). However, there was a significant inverse correlation between NLR and ChE in men (r = −0.351, *p* = 0.001), which was not significant in women (r = −0.205, *p* = 0.123). On the contrary, HPR correlated positively with ChE (r = 0.327, *p* = 0.012) in women, whereas not in men (r = 0.015, *p* = 0.885).

## 4. Discussion

Our study investigated whether a correlation can be identified between NLR, PLR, HPR and ChE in patients with LEAD attributed to a hemodynamically relevant iliac artery stenosis. The results showed a statistically significant inverse correlation between NLR and ChE, as well as a weak significant inverse correlation between PLR and ChE. Previous research has indicated that elevated levels of serum ChE are associated with poor ischemic outcomes in patients with LEAD undergoing bare metal stent implantation for SFA stenosis [26]. This is also supported by the observation of significantly lower levels of ChE in patients with critical ischemia in comparison to those with stable claudication. Moreover, numerous studies have already established an association between NLR, PLR and LEAD. For example, NLR and PLR have been identified as markers for predicting the occurrence of CLI in patients with LEAD [48,49] and have also been shown to serve as parameters for the probability of TVR following infrainguinal angioplasty [50]. In addition, NLR and PLR impact the risk of amputation and mortality in patients with acute limb ischemia [51]. Elevation of serum ChE in relation to the inflammatory parameters PLR and NLR also appears to play a role in this study population with hemodynamically relevant stenosis of the common or external iliac artery.

However, in this study, HPR showed no relevant correlation with ChE, but it has previously been brought in correlation with cancer, such as survival in patients with nasopharyngeal cancer [58], diagnostic value regarding colon cancer [59] and survival in patients with bladder cancer [60].

Two recent studies showed that PHR serves as a predictor of prognosis in patients with pulmonary embolism [52] and aortic dissection [53]. On the perspective of cardiac mortality, PHR was able to predict mortality in patients with ST-Segment Elevation Myocardial Infarction (STEMI) [55] and in patients with CAD and congestive heart failure [54].

The secondary objective of the study was to evaluate the association of inflammatory parameters, including NLR, PLR and HPR, with the quantification of stenosis, represented by δPSV. The outcome of this objective was not significant. However, other studies were able to show a significant link between LEAD and NLR or PLR [50,61,62]. A possible explanation for the counterintuitive outcome of the secondary objective could be that the pathophysiology of an elevated δPSV as documented in our study was multifactorial (e.g., primary atherosclerosis vs. in-stent restenosis) and might therefore differ from the pathophysiology of in-stent restenosis in a more heterogenous collective [50,63].

An interesting correlation emerged during the analysis of BMI and serum ChE levels. Patients with an elevated BMI showed increased levels of serum ChE. The fact that obesity is closely linked to inflammation has been widely proven in animal and human models [64,65]. It is possible that the positive correlation between BMI and serum ChE levels indicates a shared inflammatory aetiology. Ghrelin and the activated form of this peptide impact a number of obesity related pathways, including the regulation of appetite gain, energy metabolism, reward perception, the release of growth hormone and increased gastric secretion and motility [66]. However, butyrylcholinesterase is able to inactivate this peptide, by converting octanoyl-ghrelin to desacyl-ghrelin [66]. In a mouse model, it was reported that the use of galantamine, an acetylcholinesterase inhibitor in mice with diet-induced obesity, decreases the inflammatory status and reduces bodyweight and fatty liver [67]. Nevertheless, overweight (BMI 25–29.9 kg/m^2^) and moderate obesity (BMI 30–34.9 kg/m^2^) have increasingly been associated with a protective mortality benefit compared to normal BMI or more severe obesity in critically ill hospitalized and intensive care unit (ICU) patients [68]. A study was conducted with obese dogs, who were fed a high-calorie diet. The study reported a significant negative correlation between serum ChE and adiponectin concentrations [69]. Adiponectin, an anti-inflammatory hormone, being an indicator of weight gain and obesity [70], therefore may act as a link between ChE and obesity. The peptide-hormone leptin, an adipokine also being released from fatty tissue, has been brought in correlation with ChE as well. It was demonstrated that elevated leptin concentrations in adolescents with fatty liver and possibly high amounts of visceral fat significantly correlated with serum ChE levels [71].

As indicated in the introduction it appears that both high and low serum levels of ChE are potentially influencing the risk of cardiovascular disease [22,24,25]. Inflammatory mediators (TNF-α, IL-1β, IL-6, and IL-18) activate the cholinergic anti-inflammatory pathway via vagal nerve stimulation. Thereafter, an anti-inflammatory feedback mechanism is triggered by these inflammatory mediators [27]. Efferent cholinergic signaling from the vagus nerve releases acetylcholine (ACh) and inhibits via α7 nicotinic receptors on macrophages the production of proinflammatory mediators [28,29]. It thus appears reasonable to conclude, given that LEAD is an inflammatory disease [11], that elevated levels of ChE may have an impact on cardiovascular health, considering that ACh is being hydrolyzed by ChE. In another study, patients who were admitted to the intensive care unit (ICU) after cardiopulmonary bypass were analyzed in the prospect of their serum ChE levels and the development and complications of SIRS. It appeared that in patients with SIRS, ChE levels were significantly lower and that there was an association between reduced ChE levels and an increase in inflammatory cytokines, including TNF-α and IL-6 [30]. A similar observation was also reported in patients hospitalized with coronavirus disease 2019 (COVID-19) [72] which recently accounted for a significant burden especially for patients with cardiovascular diseases [73]. In 148 patients with COVID-19 an inverse correlation was documented between ChE activity and the inflammatory markers CRP and interleukin-6 [72]. Interestingly, patients hospitalized for COVID-19 with lower BMI had also lower ChE activity, which corresponds to the findings in our study showing the positive correlation of BMI and ChE [72]. Moreover, low ChE activity was associated with poor patient outcome [72]. As COVID-19 is a highly proinflammatory and prothrombotic disease [74,75,76] that may also trigger autoimmune processes [77,78] low ChE levels seem to be one further contributing factor, as they are associated with platelet reactivity [79]. The latter was shown in clopidogrel-treated patients, where patients with HRPR by the VerifyNow P2Y12 assay had lower ChE levels [79]. Though conditions of low as well as high ChE levels can be associated with poor patient outcomes, one should highlight the implications of ChE in malignant diseases and heart failure marked by low ChE levels [80,81]. Moreover, low ChE levels have also been encountered in other infectious diseases like malaria or Dengue fever [82,83]. In the latter, the administration of micronutritients like zinc was proposed to maintain ChE activity [84].

A recently published review article in 2020 highlighted the potential of acetylcholinesterase inhibitors to influence regulatory processes that upregulate cholinergic signaling and therefore accelerating anti-inflammatory pathways. Acetylcholinesterase inhibitors influencing inflammation may lead to new venues for therapeutic options in inflammatory diseases [85].

Nevertheless, the precise pathomechanism by which low levels of ChE contribute to adverse cardiovascular events, such as previously reported, remains unclear. Given its labile nature, the parasympathetic neurotransmitter ACh itself is not an optimal candidate for routine clinical measurements [86]. It was concluded that low serum levels of ChE can be interpreted as parasympathetic dysfunction, which influences adverse cardiovascular outcomes [22].

When analyzing the age difference between men and women in the study, it appeared that women were significantly older than men with a median of 70 years in women and 64 years in men. Moreover, an analysis of patient demographics indicated that individuals of advanced age were more predisposed to critical ischemia. It can be hypothesized that an influencing factor in women is the protective characteristic of estrogen [87,88] leading to a development of cardiovascular disease at a more rapid rate after menopause. This elevates the average age of being diagnosed with cardiovascular diseases in women.

A limitation of our study was its rather small sample size of 150 patients. Even after carefully selecting and ruling out patients as defined by the inclusion/exclusion criteria of this retrospective analysis, it is possible that the observed biomarkers may be influenced by undiagnosed diseases or not yet understood pathomechanisms. Moreover, we did not correct for multiple testing because our study was a pilot, exploratory study. A potential confounder is the presence of an infection among the selected patients, maybe leading to fluctuations in inflammation-dependent laboratory parameters. In addition, the age difference between stable claudication and critical ischemia can also be regarded as a confounding factor.

## 5. Conclusions

In conclusion, this study demonstrates the significant inverse correlation between NLR and serum ChE levels. Given this correlation, inflammation including ChE related pathways may be involved in the underlying pathomechanisms of LEAD. Patients with critical ischemia showed significantly lower levels of serum ChE than patients with stable LEAD further highlighting a possible link to inflammation and an adverse cardiovascular outcome. Therefore, our findings suggest that serum ChE could serve as a potential biomarker for inflammation in patients with LEAD, particularly in distinguishing between stable claudication and critical ischemia. However, further research is still needed to explore the role of ChE as a complementary biomarker, offering deeper insights into the cholinergic regulation of inflammation in LEAD.

## Figures and Tables

**Table 1:** Clinical characteristics, comorbidities, medication and Fontaine stages.

Clinical Characteristics and Comorbidities	Claudication (Fontaine II)	Critical Ischemia (Fontaine III & IV)	*p*-Value
Female/male gender [n]	46/77	12/13	0.372
Age [yrs; m (IQR)]	65 (59–70)	73 (67–81)	<0.001
BMI [kg/m^2^,m (IQR)]	26.96 (23.44–29.32)	23.11 (20.8–28.63)	0.042
Smoking [n]	116	24	1
Hyperlipidemia [n]	113	25	0.213
Type I Diabetes Mellitus [n]	1	2	0.074
Type II Diabetes Mellitus [n]	44	10	0.820
Hypertension [n]	111	20	0.168
Obesity [n]	24	3	0.570
CAD [n]	36	15	0.005
Carotid artery disease [n]	41	9	0.819
CKD [n]	12	8	0.007
COPD [n]	23	4	1
Atrial fibrillation und flutter [n]	14	4	0.509
Hypothyroidism [n]	11	1	0.691
Previous restenosis of the common or external iliac artery [n]	26	6	0.791
Previous Stroke [n]	4	3	0.094
Previous TIA [n]	1	0	1
Previous venous thrombo-embolic event [n]	4	1	1
Previous Myocardial infarction [n]	15	7	0.061
Acetylsalicylic acid [n]	99	20	1
Clopidogrel [n]	45	14	0.078
Direct oral anticoagulants [n]	8	3	0.397
Cumarine [n]	4	2	0.267
Low molecular weight heparin [n]	10	3	0.461
ACE inhibitor or ARB [n]	85	15	0.482
Ca2+ channel blocker [n]	47	9	1
Alpha-blocker [n]	14	3	1
Beta-blocker [n]	60	13	0.828
Protone pump inhibitor [n]	72	17	0.502
Lipid lowering agent [n]	106	17	0.039
Diuretics [n]	51	17	0.026
Oral antidiabetics [n]	31	7	0.804
GLP-1 receptor agonist [n]	4	1	1
Insulin [n]	16	5	0.355

ACE, angiotensin-converting-enzyme; ARB, angiotensin receptor blocker; BMI, body mass index; Ca2+, Calcium; CAD, coronary artery disease; CKD, chronic kidney disease; COPD, chronic obstructive pulmonary disease; GLP, glucagon-like peptide; IQR, interquartile- range; M, median; TIA, transient ischemic attack; yrs, years.

**Table 2:** Laboratory measurements between Fontaine stages.

Fontaine Stages	II	III	IV	III and IV	*p* (Between Fontaine Stages II and IV)	*p* (Between Fontaine Stages II and III + IV)
PLR	130.59 (93.68–169.29)	121.88 (72.07–157.32)	172.4 (123.76–251.78)	152.31 (118.21–216.96)	0.013	0.053
NLR	2.7 (2.06–3.64)	2.63 (1.33–3.53)	3.58 (2.7–6.14)	3.43 (2.51–5.45)	0.014	0.08
HPR	0.06 (0.05–0.07)	0.07 (0.04–0.08)	0.05 (0.03–0.06)	0.05 (0.03–0.07)	0.01	0.039
ChE (mg/dL)	7.76 (6.55–8.7)	7.17 (6.81–8.28)	6.33 (5.51–7.25)	6.77 (5.85–7.48)	0.001	0.004

ChE, cholinesterase; HPR, hemoglobin-to-lymphocyte ratio; NLR, neutrophile-to-lymphocyte ratio; PLR, platelet-to-lymphocyte ratio.

**Table 3:** Correlations PLR, NLR, HPR and ChE, δPSV.

Correlations	ChE	δPSV
NLR		
Correlation coefficient (r)	−0.303	−0.35
*p*-value	<0.001	0.668
PLR		
Correlation coefficient (r)	−0.162	−0.26
*p*-value	0.049	0.750
HPR		
Correlation coefficient (r)	0.122	0.081
*p*-value	0.140	0.325

ChE, cholinesterase; HPR, hemoglobin-to-lymphocyte ratio; NLR, neutrophile-to-lymphocyte ratio; PLR, platelet-to-lymphocyte ratio; δPSV, difference in peak systolic velocity.

**Table 4:** Correlations ChE and other parameters.

Correlations	ChE
δPSV	
Correlation coefficient (r)	−0.028
*p*-value	0.737
Age	
Correlation coefficient (r)	−0.122
*p*-value	0.137
BMI	
Correlation coefficient (r)	0.298
*p*-value	<0.001
Thrombocytes	
Correlation coefficient (r)	0.062
*p*-value	0.450
Leucocytes	
Correlation coefficient (r)	0.011
*p*-value	0.889
CRP	
Correlation coefficient (r)	−0.140
*p*-value	0.089
Fibrinogen	
Correlation coefficient (r)	0.069
*p*-value	0.404

BMI, body mass index; ChE, cholinesterase; CRP, C-reactive protein; δPSV, difference in peak systolic velocity.

**Table 5:** Clinical characteristics and main variables in women and men.

Clinical Characteristics and Laboratory Measures	Women (n = 58)	Men (n = 92)	*p*-Value
δPSV (m/s)	2.9 (2.02–3.5)	3.1 (2.21–4.18)	0.155
Age (yrs)	70 (64–77)	64 (59–70)	<0.001
BMI (kg/m^2^)	24.28 (21.43–29.21)	27.27 (23.83–29.18)	0.024
PLR	148.92 (104.65–191)	124.63 (94.3–161.56)	0.076
NLR	2.91 (2.26–4.06)	2.73 (2.06–4.16)	0.628
HPR	0.05 (0.04–0.06)	0.06 (0.05–0.07)	<0.001
ChE (mg/dL)	7.54 (6.53–8.44)	7.54 (6.36–8.68)	0.963

BMI, body mass index; ChE, cholinesterase; δPSV, difference in peak systolic velocity; HPR, hemoglobin-to-lymphocyte ratio; NLR, neutrophile-to-lymphocyte ratio; PLR, platelet-to-lymphocyte ratio.

## Data Availability

Data are given within the article. Further details are available on request.
